# Generation of patient specific human neural stem cells from Niemann-Pick disease type C patient-derived fibroblasts

**DOI:** 10.18632/oncotarget.19976

**Published:** 2017-08-07

**Authors:** Eun-Ah Sung, Kyung-Rok Yu, Ji-Hee Shin, Yoojin Seo, Hyung-Sik Kim, Myung Guen Koog, Insung Kang, Jae-Jun Kim, Byung-Chul Lee, Tae-Hoon Shin, Jin Young Lee, Seunghee Lee, Tae-Wook Kang, Soon Won Choi, Kyung-Sun Kang

**Affiliations:** ^1^ Adult Stem Cell Research Center, College of Veterinary Medicine, Seoul National University, Seoul 08826, Republic of Korea; ^2^ Research Institute for Veterinary Science, College of Veterinary Medicine, Seoul National University, Seoul 08826, Republic of Korea; ^3^ Institute for Stem Cell and Regenerative Medicine in Kangstem Biotech, Biomedical Science Building, Seoul National University, Seoul 08826, Republic of Korea; ^4^ Current/Present address: Hematology Branch, National Heart, Lung and Blood Institute, National Institutes of Health, Bethesda, MD 20892, USA; ^5^ Current/Present address: Biomedical Research Institute, Pusan National University Hospital, Busan 49241, Republic of Korea; ^6^ Current/Present address: Pusan National University School of Medicine, Busan 49241, Republic of Korea

**Keywords:** direct conversion, induced neural stem cell, niemann-pick disease type C, patient specific

## Abstract

Niemann-Pick disease type C (NPC) is a neurodegenerative and lysosomal lipid storage disorder, characterized by the abnormal accumulation of unesterified cholesterol and glycolipids, which is caused by mutations in the *NPC1* genes. Here, we report the generation of human induced neural stem cells from NPC patient-derived fibroblasts (NPC-iNSCs) using only two reprogramming factors *SOX2* and *HMGA2* without going through the pluripotent state. NPC-iNSCs were stably expandable and differentiated into neurons, astrocytes, and oligodendrocytes. However, NPC-iNSCs displayed defects in self-renewal and neuronal differentiation accompanied by cholesterol accumulation, suggesting that NPC-iNSCs retain the main features of NPC. This study revealed that the cholesterol accumulation and the impairments in self-renewal and neuronal differentiation in NPC-iNSCs were significantly improved by valproic acid. Additionally, we demonstrated that the inhibition of cholesterol transportation by U18666A in WT-iNSCs mimicked the impaired self-renewal and neuronal differentiation of NPC-iNSCs, indicating that the regulation of cholesterol homeostasis is a crucial determinant for the neurodegenerative features of NPC. Taken together, these findings suggest that NPC-iNSCs can serve as an unlimited source of neural cells for pathological study or drug screening in a patient specific manner. Furthermore, this direct conversion technology might be extensively applicable for other human neurodegenerative diseases.

## INTRODUCTION

Niemann-Pick disease type C (NPC) is an autosomal recessive and neurodegenerative disease caused by mutations in either the *NPC1* (95% of cases) or the *NPC2* gene (5% of cases) [1]. Over 250 different types of mutations affecting protein expression, function and stability have been identified in the *NPC1* gene. The most common mutation, *NPC1*^*I1061T*^, represents 15-20% of all disease alleles and associated with the classic juvenile-onset phenotype of NPC disease. *NPC1*^*I1061T*^ mutation disrupts NPC1 protein trafficking and mutant protein is endoplasmic reticulum (ER) retained and targeted for degradation [2]. Furthermore, defects in NPC proteins lead to impaired intracellular cholesterol trafficking, followed by the accumulation of unesterified cholesterol and sphingolipids in various organs [3]. The clinical manifestation of NPC is heterogeneous, including hepatosplenomegaly, cerebellar ataxia, and dementia, with disease onset occurring over a broad range of ages [4]. All NPC patients ultimately develop neurological symptoms, which result in disability and death, so many attempts have been made to reveal the pathogenic mechanisms [5].

NPC is currently incurable, and various reagents have been evaluated for a therapeutic potential. Cyclic oligosaccharides [6, 7], an a histone deacetylase inhibitor (HDACi) [8–10], a p38 mitogen-activated protein kinase (MAPK) inhibitor [11], a nitric oxide synthase inhibitor [12], statins [13], and rapamycin [14] have been tested for the ability to reduce the defects in neuronal functions and/or lysosomal cholesterol accumulation. These studies have primarily used several animal models [6, 7, 9, 11, 12] and human fibroblasts [8, 14] to elucidate the mechanisms underlying the therapeutic effects the investigated treatments because human brain-derived tissues or cells are inaccessible*.* Therefore, most previous studies have focused on the effects of the reagents on human fibroblasts to explain the efficacy observed in mouse models, and these findings thus cannot recapitulate the main pathologic features of human neurons. Therefore, studies utilizing disease-specific human neurons are required to provide an appropriate human pathologic model of NPC.

Recently, Bergamin et al. reported the generation of human NPC patient-derived neurons through the reprogramming of patient-derived fibroblasts [15]. However, mature neurons have little or no proliferation potential, resulting in the limited availability of large quantities of cells for high-throughput screening to evaluate drug efficacy. To this end, human pluripotent stem cell-derived neural stem cells (hPSC-NSCs) were used to understand the mechanisms of neuronal dysfunction in NPC disease [16]. That study reported that lysosomal cholesterol accumulation led to the selective neuronal defect in NSC lines generated from *NPC1* knockdown human embryonic stem cells (hESCs) because autophagy was disrupted [16]. More recently, NPC patient-derived induced pluripotent stem cells (iPSCs) were subsequently differentiated into NSCs and subsequently neurons to establish a human neuronal model of NPC disease [17–22].

Direct lineage conversion by which animal and human somatic cells can be converted into other lineage-specific cells, such as neurons [23, 24], cardiomyocytes [25], hepatic cells [26], and hematopoietic cells [27] with combinations of transcription factors has undergone rapid development. It has been reported that the combination of defined factors [28–30] or even single factor [31] can reprogram mouse or human fibroblasts into induced neural stem cells (iNSCs) with self-renewal ability. Most recently, our group reported that iNSCs with high self-renewal ability were successfully generated from various human somatic cells by forced expression of *SOX2* and *HMGA2* [32] with improved conversion efficiency. Although clinical application of neural progenitor cells derived from iPSCs or iNSCs remains elusive due to the risks associated with virus transduction, it can provide sufficient amounts of neural cells and can be used for disease modeling, such as drug screening.

Here, we report the direct reprogramming of NPC patient-derived fibroblasts into iNSCs by using defined factors. To the best of our knowledge, this is the first work that shows the generation of human iNSCs from a patient with NPC. We show that NPC-iNSCs exhibited increased cholesterol accumulation and neurological dysfunctions, recapitulating the pathological phenotype of NPC. Moreover, valproic acid (VPA) could restore defective neuronal differentiation possibly through the regulation of cholesterol homeostasis in NPC-iNSCs. Our findings demonstrate that human NPC-iNSCs obtained by direct conversion might be a promising model system in which to identify and validate candidate drugs in a human specific manner and to investigate the precise mechanisms of pathogenesis in this neurodegenerative disease.

## RESULTS

### Generation of NPC-iNSCs through direct conversion

**Figure 1:**
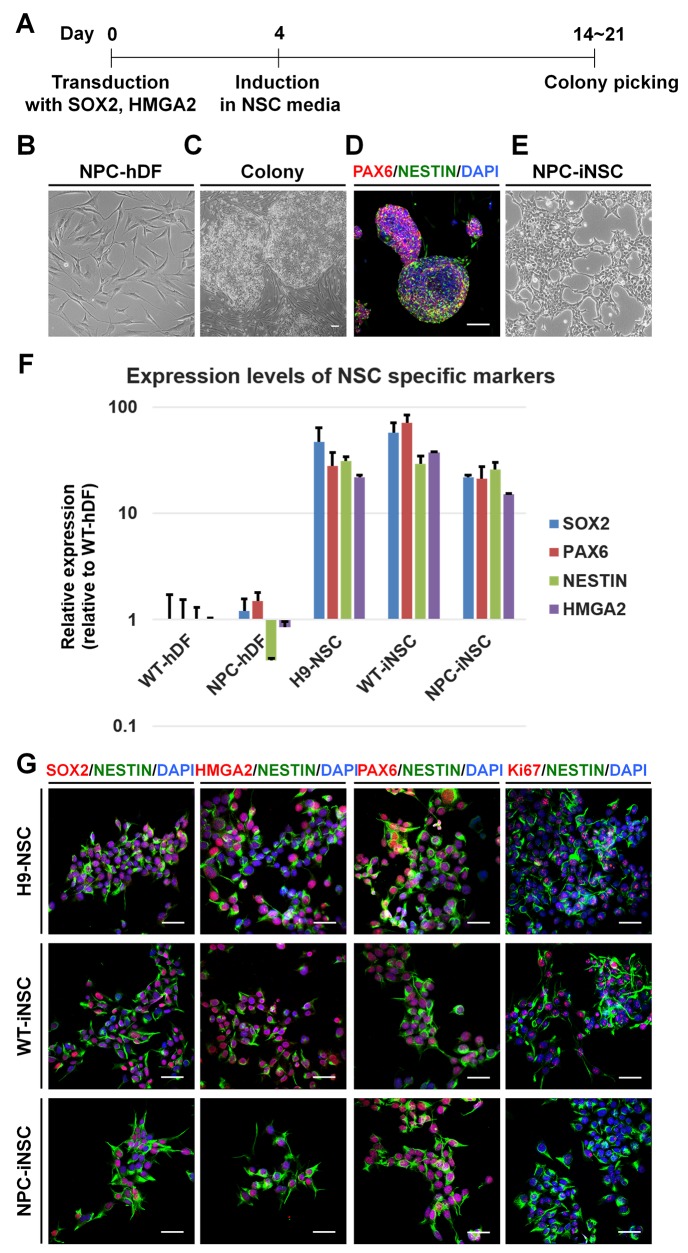
Direct conversion of NPC-iNSCs through the transduction with *SOX2* and *HMGA2* **(A)** Schematic timeline of generation of hDFs into iNSCs. (**B-E**) Shown are phase-contrast images of NPC patient-derived hDFs (B), colonies transduced with *SOX2* and *HMGA2* (C) and NPC-iNSCs (E), scale bar = 100 μm. Additionally, an immunocytochemistry analysis of colonies stained with antibodies against PAX6 and NESTIN is shown (D). Nuclei were counterstained with DAPI, scale bar = 50 μm. **(F)** Relative gene expression levels of NSC-specific markers (*SOX2*, *PAX6*, *NESTIN*, and *HMGA2*) were analyzed in NPC-hDFs, H9-NSCs, WT- and NPC-iNSCs in comparison to WT-hDFs, whose expression is considered to be 1 for all genes. **(G)** Immunocytochemistry analysis of NSC-specific marker expressions was performed in H9-NSCs, WT- and NPC-iNSCs using antibodies against SOX2, HMGA2, PAX6 and NESTIN as well as proliferating marker Ki67. Nuclei were counterstained with DAPI, scale bar = 50 μm.

After 2 or 3 weeks of iNSC induction from human dermal fibroblasts (hDFs), derived from normal donors or NPC patients, NSC-like colonies were generated (Figure 1A). The cells in these colonies exhibited a round shape with a clear border, a clearly different morphology than that of hDFs (Figure 1B and 1C), supported by immunocytochemistry using antibodies against PAX6 and NESTIN (Figure 1D). Mechanically isolated colonies were transferred to poly-L-ornithine (PLO)-and fibronectin (FN)-coated dishes and grown as a monolayer. Cells were repeatedly cultured as neurospheres and subsequently as adherent cells to obtain homogeneous iNSC lines.

### Characterization of NPC-iNSCs

NPC-iNSCs as well as WT-iNSCs showed NSC-like morphology similar to that of H9-derived human NSCs (H9-NSCs), which were used as control NSCs (Figure 1E and Supplementary Figure 1A). To identify the characteristics of generated iNSCs, we first analyzed the relative transcription levels of NSC specific markers, such as *PAX6*, *NESTIN*, *MUSASHI*, *GLAST*, and endogenous *SOX2*, *HMGA2*, in WT- or NPC-iNSCs compared to hDFs and H9-NSCs, using quantitative real-time PCR (qRT-PCR) (Figure 1F and Supplementary Figure 1B). Although NPC-iNSCs displayed slightly lower expression of *SOX2* and *HMGA2* than was observed in H9-NSCs, the expression pattern of NPC-iNSCs closely resembled that of H9-NSCs exhibiting high expression levels of NSC specific markers compared to hDFs. Thus, NSC specific markers, including endogenous *SOX2* and *HMGA2*, were successfully activated by the exogenous factor. We next performed qRT-PCR to check silencing the exogenous genes (Supplementary Figure 1C). We observed that WT- and NPC-iNSCs showed no detectable expression of retroviral specific *SOX2* and *HMGA2,* whereas WT- and NPC-hDFs on day 5 after infection exhibited high expression levels, suggesting that exogenous factors were silenced in both WT- and NPC-iNSCs. Additionally, the expression levels of several NSC specific markers were determined by immunocytochemistry (Figure 1G and Supplementary Figure 1D-1E). No obvious differences were observed in marker expression, except for the proliferation marker, Ki67. In NPC-iNSCs, Ki67 expression was dramatically lower than in H9-NSCs, suggesting that NPC-iNSCs proliferate at a relatively low rate. Furthermore, NPC-iNSCs expressed low levels of *MUSASHI* (78.8% lower than H9-NSCs) (Supplementary Figure 1B), which is also associated with NSC proliferation [33]. These results indicate that NPC-iNSCs were successfully established and highly resembled NSCs in morphology and marker expression.

### NPC-iNSCs retain disease-specific defects of NPC

**Figure 2:**
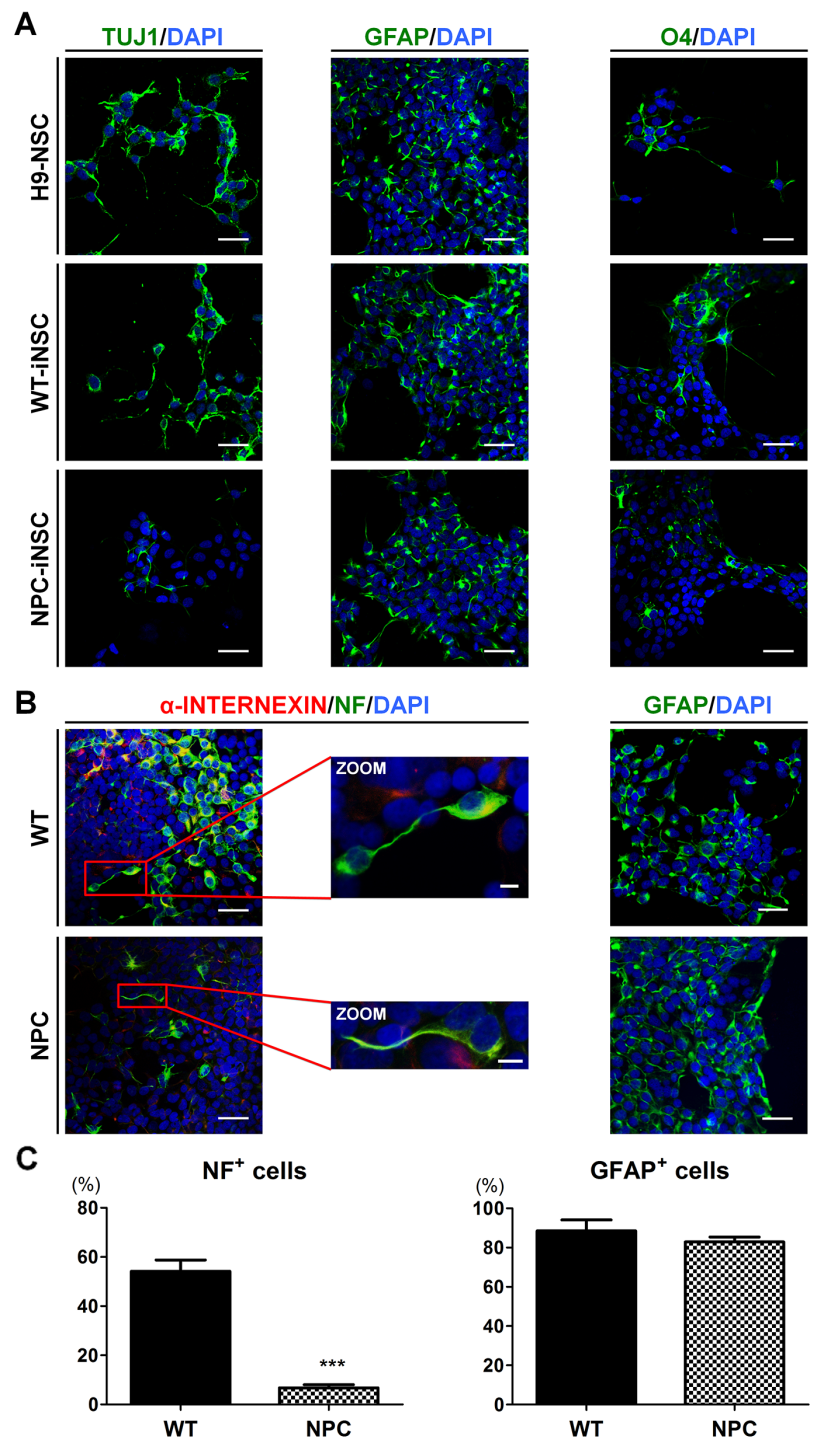
Defective neuronal differentiation of NPC-iNSCs **(A)** Representative images showing neurons, astrocytes and oligodendrocytes, differentiated from H9-NSCs, WT- and NPC-iNSCs, as indicated by TUJ1, GFAP, and O4 expression. Nuclei were counterstained with DAPI, scale bar = 50 μm. **(B)** Immunocytochemistry analysis of differentiated cells from WT- and NPC-iNSCs into neurons (α-INTERNEXIN and NF) and astrocytes (GFAP). Nuclei were counterstained with DAPI, scale bar = 50 μm, zoom scale bar = 10 μm. For the quantification in **(C)**, NF- and GFAP-positive cells were counted and were expressed as the percentage of the total number of cells, which were counterstained with DAPI, scale bar = 50 μm. ***P < 0.005.

To evaluate the multipotency of iNSCs, we examined the expression of the lineage-related markers after 7-21 days of differentiation. iNSCs showed the potential to differentiate into neuron-specific class III beta-tublin (TUJ1)-positive neurons, glial fibrillary acidic protein (GFAP)-positive astrocytes, and O4-positive oligodendrocytes (Figure 2A and Supplementary Figure 2A). These results suggested that all three types of neural lineage could be derived from iNSCs, demonstrating the multipotency of iNSCs. Interestingly, NPC-iNSCs exhibited extremely reduced levels of TUJ1 expression, whereas WT-iNSCs generated neurons with TUJ1 expression levels similar to H9-NSCs. To further evaluate whether neuronal differentiation is impaired in NPC-iNSCs, the expression levels of other neuronal markers, neurofilament (NF) and alpha-INTERNEXIN (α-INTERNEXIN), and markers of astrocyte (GFAP) were examined by immunocytochemistry (Figure 2B and Supplementary Figure 2B-2C). We observed significant decreases in the proportion of NF-positive cells, whereas the percentage of GFAP-positive cells was similar to that for WT-iNSCs (Figure 2C).

**Figure 3:**
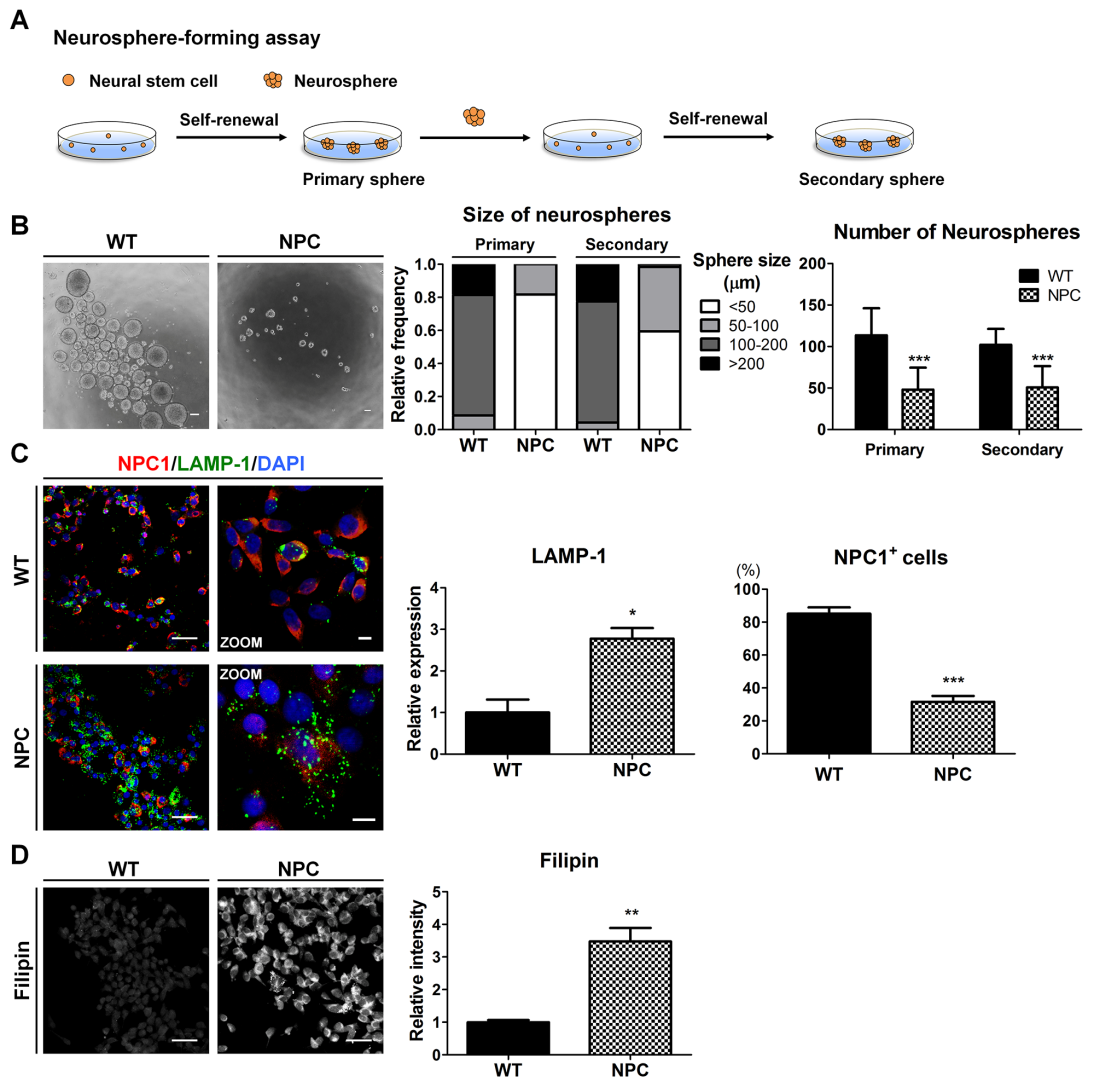
NPC-iNSCs display disease-specific phenotypes of NPC disease **(A)** Scheme of neurosphere-forming assay. **(B)** Phase-contrast images of the WT- and NPC-neurospheres (left), scale bar = 100 μm. Self-renewal ability is characterized by the diameter (middle) and number (right) of primary and secondary neurospheres. **(C)** WT- and NPC-iNSCs were stained with antibodies against NPC1 and LAMP-1. Nuclei were counterstained with DAPI,scale bar = 50 μm, zoom scale bar = 10 μm. The quantification of NPC1-postivie cells was conducted following the same method used in Figure 2C. The expression levels of LAMP-1 was measured using Image J software and the value of control is set at 1. **(D)** Unesterified cholesterol of WT- and NPC-iNSCs was detected by filipin staining, scale bar = 50 μm. *P < 0.05, **P < 0.01, ***P < 0.005.

Given that the self-renewal capacity of NSCs is crucial for their multipotent differentiation [11], we next investigated the self-renewal of NPC-iNSCs. For the neurosphere-forming assay, 1,000-2,000 cells were plated on non-adherent 24-well culture dishes to form primary neurospheres. Primary neurosphere cells were dissociated into single cells using Accutase and were subsequently replated at a clonal density in non-adherent cultures to form secondary neurospheres (Figure 3A). NPC-neurospheres were markedly different in their size and number compared to WT-neurospheres (Figure 3B and Supplementary Figure 3A). NPC-iNSC-derived neurospheres were observed primarily as smaller neurospheres (< 50 μm), whereas WT-iNSC-derived neurospheres accumulated as large neurospheres (> 100 μm). Furthermore, significantly fewer neurospheres were derived from NPC-iNSCs than from WT-iNSCs.

NPC1 protein with I1061T mutation represents misfolded NPC1 protein, which is targeted for ER-associated degradation [2]. NPC1-mutated cells displayed high expression levels of lysosomal-associated membrane protein (LAMP), a regulator of lysosomal membrane stability [34]. We found that NPC-iNSCs exhibited fewer NPC1-positive cells and higher expression level of LAMP-1 compared to WT-iNSCs (Figure 3C and Supplementary Figure 3B). Higher LAMP-1 expression with mutated NPC1 in NPC-iNSCs indicates the accumulation of unesterified cholesterol in lysosomes due to defective transportation [2, 4]. To investigate whether the pathogenic phenotypes in NPC could be replicated in NPC-iNSCs, we evaluated the cholesterol accumulation levels in WT- and NPC-iNSCs using filipin staining, a general tool for the detection of free cholesterol in cells (Figure 3D and Supplementary Figure 3C). We found that NPC-iNSCs strongly expressed filipin similar to NPC-hDFs, whereas WT-iNSCs and WT-hDFs showed low filipin expression (Supplementary Figure 3D). Therefore, these data revealed that NPC-iNSCs consistently retain the phenotype of NPC-hDFs. In addition, the accumulated cholesterol in iNSCs and hDFs was quantified using a cholesterol assay kit. NPC-iNSCs and NPC-hDFs displayed extensive intracellular cholesterol accumulation compared with control iNSCs and hDFs (Supplementary Figure 3E). Taken together, these results indicate that NPC-iNSCs recapitulate the main features of pathological status in NPC.

### Inhibition of cholesterol transport leads to impaired capacities of self-renewal and neuronal differentiation in WT-iNSCs

**Figure 4:**
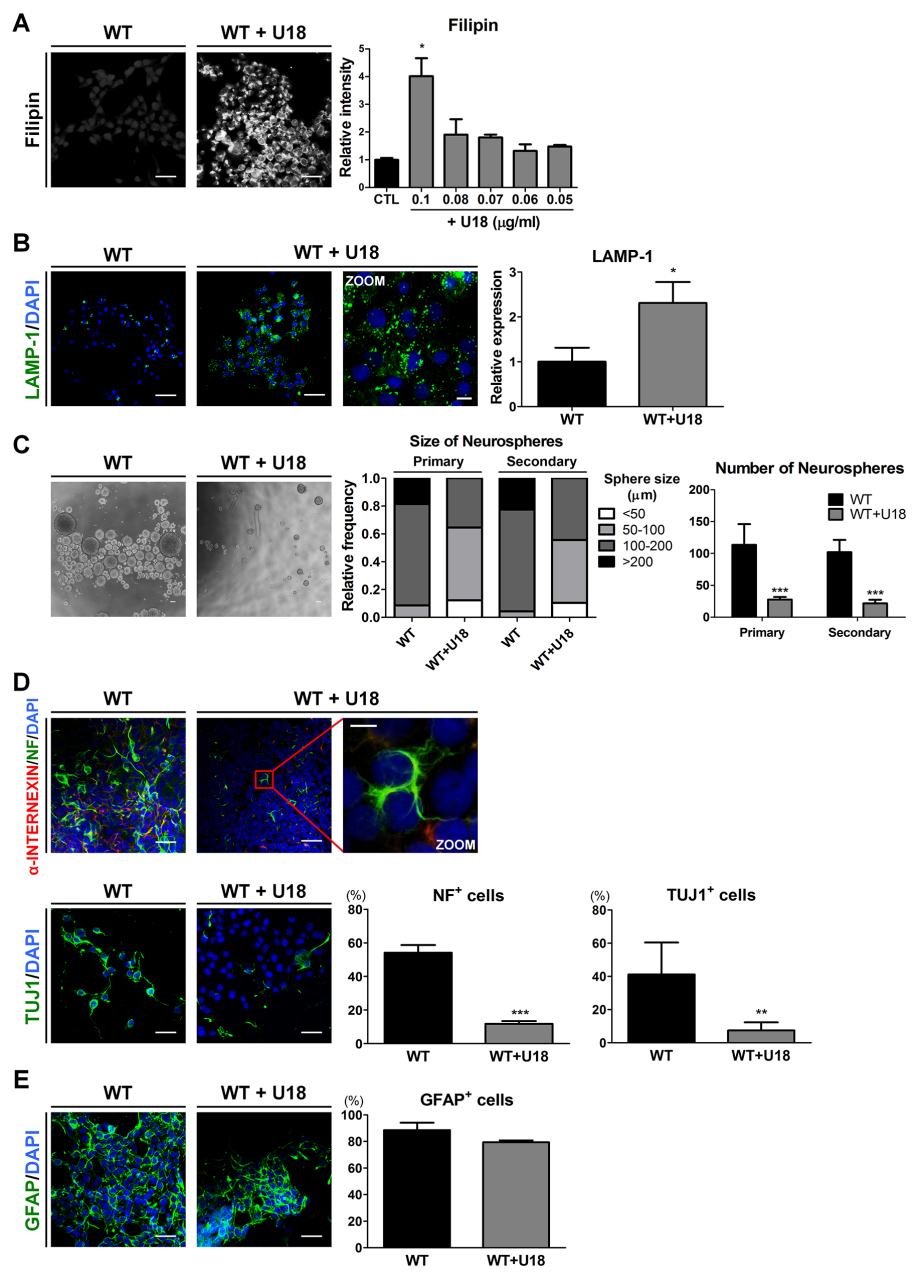
U18 treatment leads to impairments of self-renewal and neuronal differentiation in WT-iNSCs through abnormal cholesterol accumulation **(A)** Representative filipin staining results indicates that U18 treatment induced cholesterol accumulation, compared to non-treated WT-iNSC, scale bar = 50 μm. U18 was treated at various concentration to determine the appropriate concentration. The intensity of filipin was analyzed and quantified. **(B)** U18-treated WT-iNSCs were stained with antibody against LAMP-1 and quantified using the same method as in Figure 3C, scale bar = 50 μm, zoom scale bar = 10 μm. **(C)** Phase-contrast images of the control- and U18-treated WT-neurospheres, scale bar = 100 μm (left). Self-renewal ability of U18-treated WT-neurosphere was characterized by the size (middle) and number (right) of neurospheres. **(D)** After U18 treatment, WT-iNSCs were differentiated into neurons (α-INTERNEXIN, NF, and TUJ1). Nuclei were counterstained with DAPI, scale bar = 50 μm, zoom scale bar = 10 μm. **(E)** U18-treated WT-iNSCs and non-treated WT-iNSCs were differentiated into GFAP-positive astrocytes. Nuclei were counterstained with DAPI, scale bar = 50 μm. Differentiation capacity into neurons **(D)** and astrocytes **(E)** was quantified using the same method as in Figure 2C. *P < 0.05, **P < 0.01, ***P < 0.005.

To determine whether the accumulation of unesterified cholesterol leads to the defective abilities of self-renewal and neuronal differentiation, we used amphiphilic amino-steroid U18666A (U18), an inhibitor of intracellular cholesterol trafficking [35], to mimic the cholesterol transport impairment of NPC in WT-iNSCs and WT-hDFs. First, to confirm the cytotoxicity of U18, the cells were exposed for 24 hours to different concentrations ranging from 1 to 0.05 μg/ml of U18 to WT-iNSCs and 4 to 0.1 μg/ml of U18 to WT-hDFs (Supplementary Figure 4A). U18 concentrations from 0.05 to 0.1 μg/ml in WT-iNSCs and 0.1 to 1 μg/ml in WT-hDFs showed no significant evidence of cytotoxic damage. Concentration of U18 more than 0.2 μg/ml in WT-iNSCs and 2 μg/ml in WT-hDFs caused a notable increase in cell cytotoxicity. We also used filipin staining to explore the dose-dependent effects of U18 in order to determine the appropriate concentration (Figure 4A and Supplementary Figure 4B-4C). Treatment with 0.1 μg/ml U18 in WT-iNSCs and 1 μg/ml U18 in WT-hDFs for 24hrs resulted in dramatic increases in cholesterol accumulation compared to the control, without notable cytotoxicity. In addition, U18-treated WT-iNSCs exhibited increased expression level of LAMP-1 (Figure 4B). Next, we performed the neurosphere-forming assay to examine the self-renewal ability of U18-treated WT-iNSCs (Figure 4C). Surprisingly, only 40% of neurospheres derived from U18-treated WT-iNSCs reached more than 100 μm in diameter, compared with > 70% of WT-neurospheres. The average number of U18-treated WT-neurospheres was significantly lower than from control WT-neurospheres. These findings were comparable with NPC-neurospheres, implying that defective self-renewal ability may result from abnormal cholesterol accumulation.

To identify the effect of cholesterol accumulation on neurogenic potential, we differentiated WT-iNSCs into neurons and astrocytes after U18 treatment. We found that neuronal cells derived from U18-treated WT-iNSCs displayed a significant decrease in NF- and TUJ1-positive cells compared to control WT-neurons (Figure 4D), whereas there were no discernable differences in GFAP expression (Figure 4E). These findings suggest that inhibition of cholesterol trafficking can hamper the abilities of normal iNSCs to self-renew and to differentiate into neurons, similar to the behavior observed for NPC-iNSCs. Taken together, these data suggest that regulation of cholesterol homeostasis might be an essential mechanism for governing self-renewal and differentiation in NPC.

### Effects of VPA on cholesterol accumulation and self-renewal ability in NPC-iNSCs

**Figure 5:**
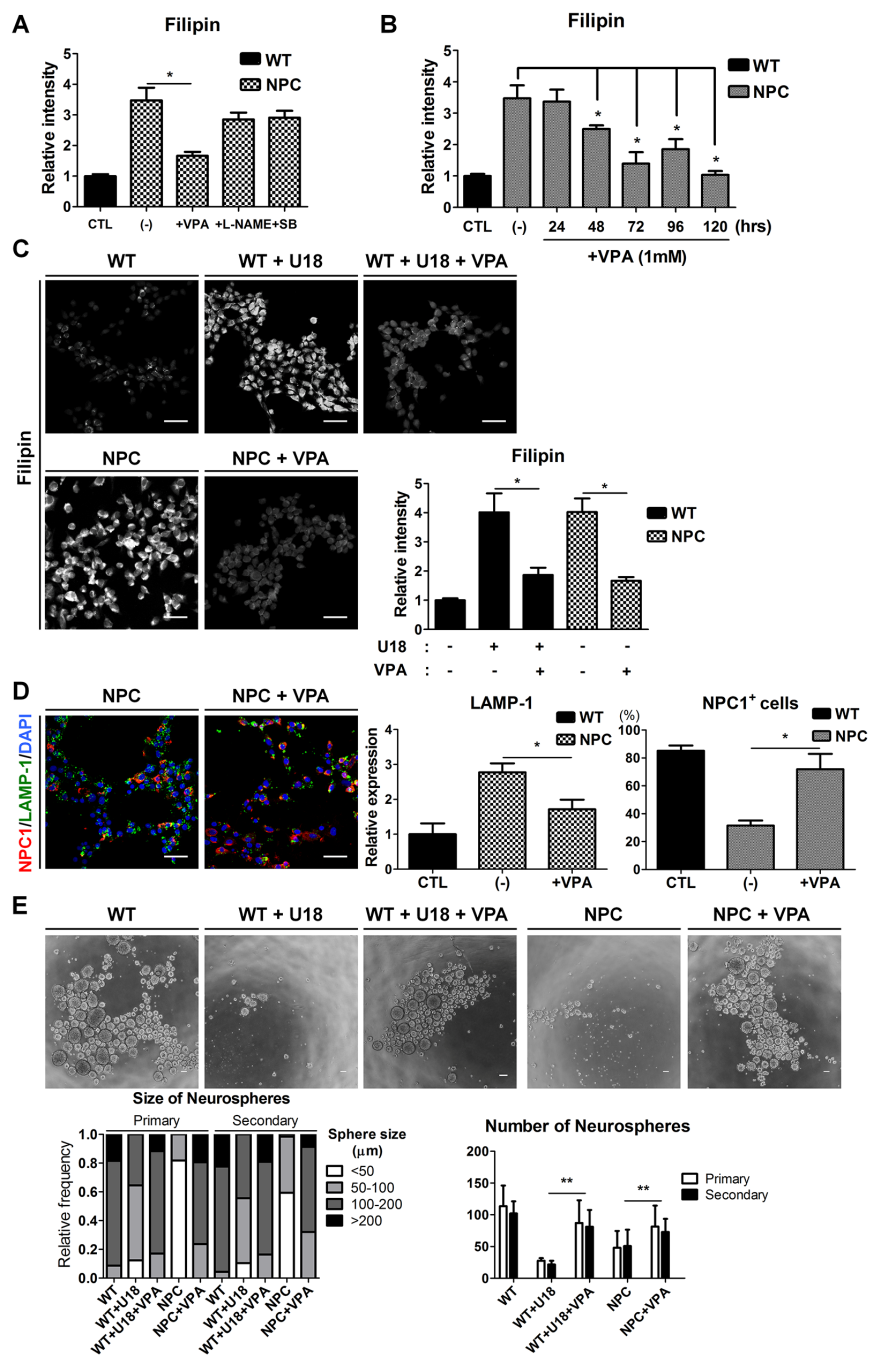
Rescue of cholesterol accumulation and self-renewal ability by VPA treatment **(A)** NPC-iNSCs were treated with VPA (1mM), L-NAME (100μM), and SB202190 (1μM) for 3 days. **(B)** VPA was treated to NPC-iNSCs at various incubation time. The relative intensity of filipin was analyzed and normalized to WT-iNSCs. **(C)** VPA treatment had effect on reduction of cholesterol accumulation in U18-treated WT- and NPC-iNSCs, scale bar = 50 μm. **(D)** VPA-treated NPC-iNSCs were stained with NPC1 and LAMP-1, scale bar = 50 μm. The quantification in (D) was conducted following the same method as in Figure 3C. **(E)** Analysis of neurosphere formation after VPA treatment. Impaired self-renewal ability was rescued by VPA treatment, scale bar = 100 μm. *P < 0.05,**P < 0.01.

To evaluate the potential of NPC-iNSCs as a drug-screening platform, we treated these cells with therapeutic compounds. Our previous studies have shown that NSCs from NPC1^-/-^ mice showed restored self-renewal after the inhibition of p38 MAPK signaling [11] and showed improved functions through the control of abnormal NO-mediated signaling by *N*^*ω*^-nitro-L-arginine methyl ester (L-NAME) [12]. In addition, VPA has been reported as an effective drug to decrease cholesterol accumulation in NPC [8–10, 36]. Based on previous studies, we tested the candidate drugs SB202190 (1 μM), L-NAME (100 μM), and VPA (1 mM) on NPC-iNSCs and confirmed their effects on the regulation of cholesterol accumulation using filipin staining (Figure 5A and Supplementary Figure 5A). Interestingly, the filipin staining revealed that cholesterol accumulation was drastically reduced only in VPA-treated cells, whereas other compounds exhibited slight but not significant decreases in filipin staining. Thus, we focused on the effect of VPA treatment on NPC-iNSCs and subsequently explored the optimal conditions for the VPA treatment (Figure 5B and Supplementary Figure 5B). We observed significant decreases in cholesterol accumulation, evidenced by filipin staining, in the VPA-treated groups (Figure 5C and Supplementary Figure 5C). Previous report showed HDACi treatment in NPC patient fibroblasts partially restored NPC1 expression [8]. As expected, VPA-treated NPC-iNSCs displayed increased NPC1-positive cells (Figure 5D). In addition, we explored that expression levels of LAMP-1 in VPA-treated NPC-groups (iNSCs and fibroblasts) were significantly decreased (Figure 5D and Supplementary Figure 5D). These findings imply that restoration of cholesterol accumulation were resulted from partially rescued NPC1 protein. Moreover, these results indicate that VPA can rescue not only the cholesterol accumulation in NPC but also the abnormal cholesterol accumulation induced by U18 treatment. Additionally, we confirmed that the effect of VPA on the cholesterol accumulation in NPC-iNSCs and NPC-hDFs was consistent by detecting cholesterol levels (Supplementary Figure 5E).

To assess the effect of VPA in other dysfunctions of NPC-iNSCs, we performed neurosphere-forming assays with VPA treatment (Figure 5E). We measured the size and number of neurospheres from U18-treated WT-iNSCs and NPC-iNSCs after VPA treatment (1 mM for 72hrs). VPA treatment rescued the defective neurosphere-forming ability in NPC-iNSCs, as well as in U18-treated WT-iNSCs. VPA-treated cells primarily formed large neurospheres (> 100 μm), with some even reaching > 200 μm in diameter. The average number of U18-treated WT- and NPC-neurospheres was markedly increased after VPA treatment. Taken together, these results indicate that VPA restores, at least partly, the abnormal cholesterol accumulation and impaired self-renewal ability.

### VPA-induced rescue of the neuronal differentiation of NPC-iNSCs may occur via the regulation of cholesterol metabolism

**Figure 6:**
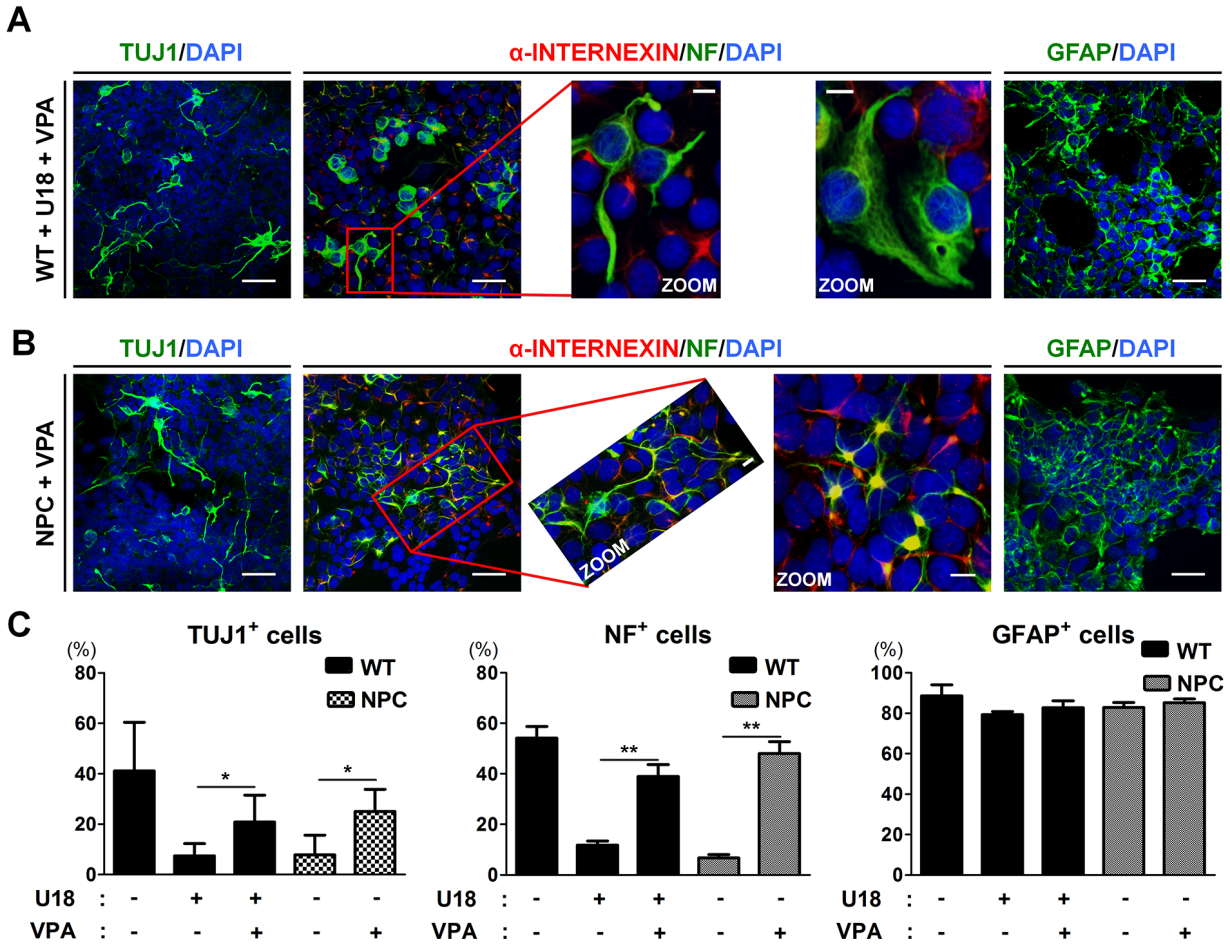
VPA treatment partially rescues the neuronal differentiation in NPC-iNSCs **(A-B)** Representative images of α-INTERNEXIN-, NF-, TUJ1-, and GFAP-positive cells differentiated from U18-treated WT-iNSCs (A) and NPC-iNSCs (B) after VPA treatment, scale bar = 50 μm, zoom scale bar = 10 μm. **(C)** The quantification was conducted following the same protocol of Figure 2C. *P < 0.05, **P < 0.01.

To identify the impact on neurogenic potential of the VPA-induced reduction in cholesterol level, we differentiated U18-treated WT-iNSCs or NPC-iNSCs into neurons with VPA treatment at 1 mM every other day. Following VPA treatment, we found that the number of TUJ1- and NF-positive cells was significantly increased from U18-treated WT- and NPC-iNSCs compared to non-treated WT-cells (Figure 6A-6C). In addition, NPC-iNSCs derived from multiple donors displayed consistent restoration in their ability to differentiate into neurons after VPA treatment (Supplementary Figure 6A). The astrocyte differentiation capability of U18-treated WT- and NPC-iNSCs was not altered by VPA treatment. These results suggests that VPA pretreatment promotes differentiation into neurons in NPC-iNSCs.

Next, we screened cholesterol metabolism-related genes in WT-iNSCs, NPC-iNSC, and VPA-treated NPC-iNSCs using qRT-PCR (Supplementary Figure 6B). Liver X receptor *β* (*LXR β)* activation has been reported to inhibit the accumulation of excess cholesterol and to stimulate cholesterol efflux through the induction of transcription of downstream genes, such as *SREBPs* (sterol regulatory element-binding proteins) and *ABC* (ATP-binding cassette transporter) [37]. Therefore, we measured the expression of these downstream genes. The expression level of *SREBP1* and *SREBP2*, which enhances the transcription of various genes required for cholesterol synthesis [37, 38], was considerably reduced after VPA treatment. In contrast, *ABC isoform A1* (*ABCA1*) and *G1* (*ABCG1*), known as a cholesterol transporter gene, was markedly up-regulated by VPA treatment, indicating that excess cholesterol is exported normally. Taken together, our results demonstrate that VPA rescues the abnormal neuronal differentiation of NPC-iNSCs possibly via the *LXR β*–mediated regulation of cholesterol homeostasis.

## DISCUSSION

In the present study, we show direct reprogramming of NPC patient-derived fibroblasts into iNSCs using only two factors, *SOX2* and *HMGA2*. We previously demonstrated that *HMGA2* facilitated the efficient reprogramming of senescent somatic cells or CD34-positive blood cells toward iNSCs through synergistic interactions with *SOX2* [39]. Here, we successfully established iNSC lines from NPC patient fibroblasts using our optimized reprogramming factors and protocols.

Established NPC-iNSCs showed no obvious differences from H9-NSCs and WT-iNSCs in terms of their morphology and NSC-specific marker gene expression. Although NPC-iNSCs showed decreased proliferation compared to WT-iNSCs, they were expandable, suggesting that the cells can be stably maintained. Similarly, previous studies reported that NPC patient-specific iPSCs and subsequently differentiated NSCs showed morphology and gene expression profiles nearly identical to those of their wild-type counterparts [23–25]. Each NPC-iPSCs or NPC-iNSCs has pros and cons for disease modeling. iPSCs are well-characterized cell type for a decade of vigorous research after its first establishment. Furthermore, once NPC disease-specific iPSCs are established, its unique pluripotent potential provides the opportunity to differentiate into different cell type, such as hepatic cells, another affected cell type in NPC disease [20]. On the other hands, iNSCs are relatively newly established cell type [28, 29, 31, 32], requiring further validation of defined factors to generate iNSCs, clonality, or iNSC state. Recent report suggested that clonal iNSC lines from distinct genetic backgrounds influence the cell fate transition into an iNSC state [40]. However, a direct reprogramming strategy of iNSCs would bypass the risk of the intermediate pluripotent state, thus avoiding the potential contamination risk of pluripotent cells [41]. Development of clonally derived, clinical-grade iNSCs (e.g. non-viral method) warrant further studies for the future application of the genetically/chemically corrected NPC-iNSCs in the clinical setting.

NPC includes various clinical manifestations, but death of NPC patients is significantly correlated with the progression of neurodegeneration [4], suggesting that it is essential to establish a neuronal model of NPC for elucidating the pathogenic mechanisms. NPC-iNSCs can be generated to establish an appropriate cellular model system for the neurological study of NPC through drug screening, toxicity testing, or cell transplantation. Therefore, it is critical for NPC-iNSCs to retain the disease-specific defects of NPC. We found that increased cholesterol accumulation and defective self-renewal were consistently maintained in NPC-iNSCs and their progeny. These findings suggested that iNSCs may adequately reflect the NPC disease phenotype. Although NSCs or neural progenitor cells from NPC-iPSCs have suggested the controversial interpretation regarding disease-relevant defects, such as in cholesterol metabolism and autophagic flux [17, 18, 20], these studies showed clear defects in NPC-iPSC-derived neuronal cells. Further studies need to be done to elucidate disease-relevant defects in NPC-iNSC derived neuronal cells including apoptosis and autophagy. Furthermore, we induced impaired intracellular cholesterol trafficking in WT-iNSCs by U18 treatment to mimic the NPC phenotype. U18 blocks the cholesterol export out of lysosomes and can directly bind to a site that is within a section of the NPC1 protein, sterol-sensing domain [42], suggesting it precisely targets NPC1 in WT-iNSCs. U18-treated WT-iNSCs displayed abnormal cholesterol accumulation, defective neurosphere-forming ability, and impaired neural differentiation, similar to NPC-iNSCs. These results indicated that the regulation of cholesterol efflux system plays an important role in the pathological phenotypes of NPC disease.

To validate NPC-iNSCs as a promising tool for the screening of therapeutic compounds, we applied VPA treatment based on our previous data [9]. Many studies have reported that VPA enhances neural differentiation of various cell types, such as adult neural progenitor cells from rats [43], adult hippocampal neural progenitor cells of rats [44], and sympathoadrenal progenitor cells from cattle [45]. However, the effects of VPA on NPC-patient derived iNSCs had not yet been elucidated. Our observations confirm that VPA treatment reduced cholesterol accumulation and restored not only self-renewal ability but also neuronal differentiation in U18-treated WT-iNSCs and NPC-iNSCs. Furthermore, we screened several genes related to cholesterol metabolism and found that VPA regulated *LXR β*, known as a cholesterol sensor, and its downstream genes in NPC-iNSCs. These findings suggest that VPA may have restored the defective neuronal differentiation of NPC-iNSCs via regulating cholesterol homeostasis. However, the concentration of VPA in current study (1mM) could regulate several nonhistone proteins, transcription factors, cytoskeletal proteins, and molecular chaperones [8, 46, 47]. Because chaperone activity-mediated up-regulated NPC1 mutant protein can reverse the NPC phenotype in NPC1^I1061T^ mutant human fibroblast cell lines [2], further studies are required to elucidate the details of other modes of action of VPA in NPC-iNSCs.

Here, for the first time, we generated iNSCs from NPC patient-derived fibroblasts. Established NPC-iNSC lines show self-renewal ability and can be expanded sufficiently *in vitro*, suggesting that these cell lines can provide sufficient amounts of patient-specific cells for various therapeutic studies including drug screening. These disease-specific neural cells exhibit the NPC phenotypes of cholesterol accumulation and defective self-renewal and neuronal differentiation, which can be partially corrected by VPA treatment. Because NPC-iNSCs can reproduce the features of a human lysosomal storage disorder, and may further reflect the biochemical and physiological defects of patients, we expect that NPC-iNSCs as a model system provide an excellent strategy for the development of clinical treatments for NPC.

## MATERIALS AND METHODS

### Generation of iNSCs through direct conversion

iNSCs were generated from normal donor skin fibroblasts (GM05659; Coriell Institute for Medical research, Camden, NJ, USA) and NPC patient-derived hDFs (GM03123 (*NPC1*^P237S/I1061T^), GM18453 (*NPC1*^I1061T/I1061T^); Coriell Institute for Medical research). Viral production and transduction were performed as described previously [32]. Briefly, the retroviral pMX-SOX2 and pMX-HMGA2 were transfected into 293 FT cells along with VSV-G and gag/pol plasmids using Fugene 6 transfection reagent (Roche, Indianapolis, IN, USA). The viral supernatants were collected at 48 and 72 hours post-transfection and used to infect hDFs with 5 μg/ml polybrene (Sigma-Aldrich, Sigma, St. Louis, MO, USA). For neural stem cell induction, medium was changed to the NSC maintenance medium (ReNcell NSC maintenance media; Millipore, Billerica, MA, USA) with basic fibroblast growth factor (bFGF; Sigma) and epidermal growth factor (EGF; Sigma) after expansion of the infected cells. NSC-like colonies were picked and cultured in neurosphere culture condition. To generate a homogenous population of iNSCs, cells were maintained as neurosphere and cultured as attached cells on PLO/FN-coated dishes, repeatedly. NPC-iNSC lines from NPC1 mutant human fibroblast were generated up to 10 independent clones. Representative images shown in main figures were from GM03123-derived NPC-iNSCs. Replicated results using different clones of GM03123 (#3) and GM18453 (#12) were shown in Supplementary Figures 1-6.

### *In vitro* differentiation

iNSCs at 5,000 cells per well were seeded onto PLO/FN-coated coverslips in 24-well plates, and ReNcell NSC maintenance media (Millipore) with GlutaMAX (GMAX; Thermo Fisher Scientific, Thermo, Waltham, MA, USA) was added for random differentiation. After 2days of random differentiation, the medium was replaced for the induction of three specific lineages (neurons, astrocytes, and oligodendrocytes). The induction media were prepared and used as previously described [32]. Briefly, neurons were generated in a neuron differentiation medium containing a 1:1 mixture of Neurobasal medium (Gibco BRL, Gibco, Grand Island, NY, USA) and DMEM/F12 medium supplemented with B27 (Gibco), GMAX, retinoic acid (RA) (Sigma), ascorbic acid (Sigma), brain-derived neurotrophic factor (BDNF; Peprotech, Rocky Hill, NJ, USA), glial-cell-line-derived neurotrophic factor (GDNF; Peprotech), and forskolin (Sigma). The astrocyte induction medium was composed of DMEM (high glucose) with N2 (Gibco), GMAX, and 1% FBS. For oligodendrocyte induction, the medium containing DMEM/F12 with N2, MEM nonessential amino acids solution (MEM NEAA; Gibco), heparin (Sigma), RA, sonic hedgehog (SHH; Peprotech), and B27. After 2 weeks, the induction medium was changed to DMEM/F12, supplemented with N2, B27, MEM NEAA, T3, cyclic AMP (cAMP; Sigma), platelet-derived growth factor (PDGF; Peprotech), insulin-like growth factor (IGF; R&D Systems, Minneapolis, MN, USA), and neurotrophin-3 (Sigma).

### Immunocytochemistry

Cells were washed 3 times with phosphate buffered saline (PBS; Gibco) and fixed with 4% paraformaldehyde (PFA) in PBS for 10 minutes at room temperature. Fixed cells were then permeabilized with 0.3% Triton X-100 for 15 minutes and were incubated with blocking solution containing 5% normal goat serum (NGS; Zymed, San Francisco, CA, USA) for 1 hour at room temperature. Primary antibodies were used in blocking solution according to the manufacturer’s recommended dilution, and cell treated with primary antibody were incubated overnight at 4°C. Cell incubation with secondary Alexa 488- or Alexa 594-labeled antibodies (Molecular Probes, OR, USA) was performed for 1 hour at room temperature. For nuclei staining, 4’, 6-diamidino-2-phenylindole (DAPI; Sigma) was used for 10 minutes. Images were captured on a confocal microscope (Nikon, Eclipse TE200, Japan).

### Filipin staining

Cells were fixed with 4% paraformaldehyde for 10 minutes at room temperature. Fixed cells were boiled with antigen retrieval citrate buffer (10 mM Sodium citrate, 0.05% tween 20, pH 6.0) at 85°C for 10 minutes. Subsequently, cells were incubated with 100 μg/ml filipin (Cayman, Ann Arbor, MI, USA) in PBS for 1 hour. Images were captured by confocal microscope (Nikon). The density of filipin staining in the cytoplasm was quantified using Image J software as previously reported [48]. More than 5 fields were analyzed for each condition and the graph represent the average of the quantification. The value of control is standardized as 1.

### Statistical analysis

All experiments were conducted at least three times by using different cell lines established from NPC patient skin-derived fibroblast cell lines; GM03123 (*NPC1*^*P237S/I1061T*^), GM18453 (*NPC1*^*I1061T/I1061T*^) or normal human skin fibroblasts; GM05659. All of the statistical comparisons were conducted via two-tailed Student’s t-test or one-way ANOVA followed by Bonferroni post-hoc test for multigroup comparisons using GraphPad Prism version 5.0 (GraphPad Software, San Diego, CA, USA). Statistical significance is indicated in the figure legends.

### Supplementary information

Supplemental materials provide supplemental experimental procedures including cell culture, RT-PCR, neurosphere formation assay, chemical treatment, cholesterol assay and cytotoxicity assay.

## SUPPLEMENTARY MATERIALS FIGURES


