# Supplementary material for: Generation of patient specific human neural stem cells from Niemann-Pick disease type C patient-derived fibroblasts
Source: Oncotarget. 2017 Aug 7;8(49):85428–41. doi: 10.18632/oncotarget.19976 (PMC5689620; doi:10.18632/oncotarget.19976)
Supplement: Supplementary file 1 [file oncotarget-08-85428-s001.pdf]

Generation of patient specific human neural stem cells from Niemann-Pick disease type C patient-derived fibroblasts

SUPPLEMENTARY MATERIALS

Cell culture

NPC patient skin-derived fibroblast cell lines (NPC fibroblast; GM03123 (*NPC1*$^{P237S/I1061T}$) and GM18453 (*NPC1*$^{I1061T/I1061T}$)) and normal donor skin fibroblasts (GM05659) were purchased from the Coriell institute for Medical research (Camden, NJ). hDFs were cultured in fibroblast growth medium-2 (Gibco) containing 10% fetal bovine serum (FBS; Gibco). H9-derived human neural stem cells (H9-NSCs; NA800-100) were purchased from Gibco and maintained in NSC maintenance medium (ReNcell NSC maintenance media; Millipore) with bFGF (Sigma) and EGF (Sigma).

Reverse transcription polymerase chain reaction (RT-PCR) and real-time PCR

RT-PCR and real-time PCR were performed as previously described [1]. Briefly, total cellular RNA was extracted from the cells using TRIzol reagent (Invitrogen, Carlsbad, CA) according to the manufacturer's instructions. Purified RNA was transcribed to complementary DNA using Super-script III First-Strand Synthesis System (Invitrogen). PCR was performed using SYBR Green PCR Master Mix (Applied Biosystems, Foster City, CA) with each primer. To quantify gene expression, ABI 7300 sequence detection system with supplied software (Applied Biosystems) was used. Each gene was normalized with *GAPDH* as a housekeeping control, and gene expression levels were measured at least three independent analyses.

Neurosphere formation assay

On non-adherent culture dishes, 2,000 cells were cultured to form primary neurospheres. This experiment was performed in triplicate. The number and size of primary neurospheres were measured 7 days later. Subsequently, primary neurospheres were dissociated into single cell suspension with accutase (Gibco), then replated at clonal density (2,000 cells per well of 24-well plate) on non-adherent dishes. Secondary neurospheres were generated in triplicate and counted 7 days later to quantify the number of secondary neurospheres formed per primary neurospheres. Individual neurospheres were transferred to adherent plates to allow spheres to evaluate differentiation potentials.

Chemical treatment

The effect of drugs on the cholesterol accumulation was evaluated by treating VPA (1 mM; Sigma), SB202190 (1 µM; Millipore), and L-NAME (100 µM; Tocris Bioscience) into NPC-iNSCs. Each drugs were treated per day, at least three times.

Cholesterol assay

For sample preparation, 1 x 10^6 cells were trypsinized and treated with 200 µl of cholesterol lysis buffer (chloroform: isopropanol: NP-40 (7:11:0.1)). The samples were incubated in 50°C oven to remove chloroform overnight, and then dried samples were dissolved in 200 µl of cholesterol lysis buffer by sonication. The cholesterol standard in cholesterol assay kit (Bio Vision, Milpitas, CA) was generated by adding 20 µl of the cholesterol standard to 140 µl of cholesterol assay buffer, mixed well, and added 0, 4, 8, 12, 16, and 20 µl into different wells. Subsequently, reaction reagents were mixed enough for the number of assays (samples and standards) and incubated for 1 hour at 37°C, protected from light. The absorbance of the assays was measured at 570nm in a microplate reader.

Cytotoxicity assay

The WT-cells were treated with U18666A (U18) in a various concentration range (1 – 0.05 µg/ml). To measure cytotoxicity, MTT assay was conducted. After treatment for 24 hours, cells were incubated in fresh medium containing 200 µg/ml of MTT reagent (Amresco, OH, USA) for 4 hours at 37°C with 5% CO_2. After removal of medium, DMSO was put into each well and incubated with shaking for approximately 2~3 min. The results were measured by an ELISA reader. Empty wells were blank controls and untreated cells were used as controls. Cell cytotoxicity determined by MTT assay was expressed as percent of dead cells.

REFERENCE

1. Yang SR, Jung JW, Kim H, Ko K, Han DW, Park SB, Choi SW, Kang SK, Schöler H, Kang KS. CD49f enhances multipotency and maintains stemness through the direct regulation of OCT4 and SOX2. Stem Cells. 2012; 30:876-887.

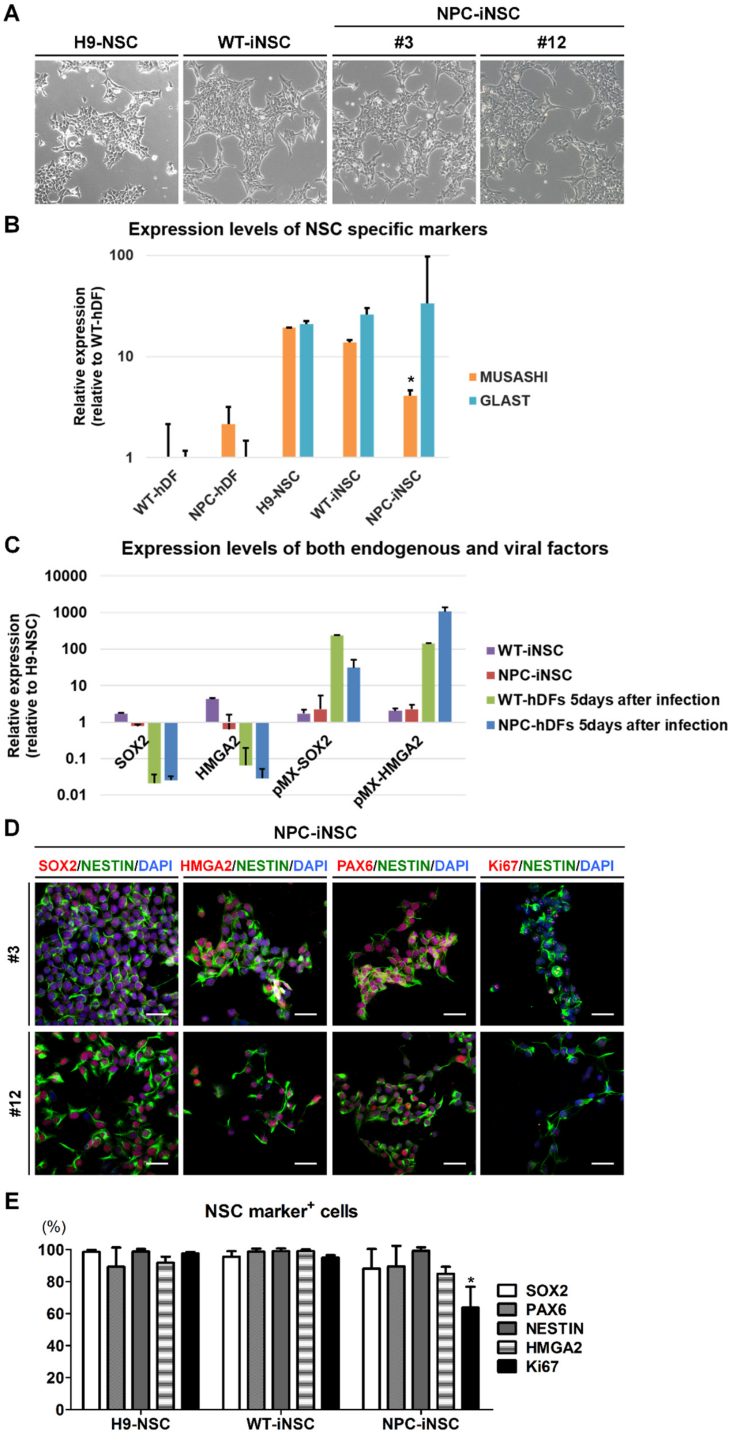

Supplementary Figure 1: Characterization of NPC-iNSCs. (A) Phase-contrast images of H9-NSC, WT- and NPC-iNSCs (#3 and #12), scale bar = 100 µm. **(B)** Relative expression levels of NSC-specific marker genes (*MUSASHI* and *GLAST*) were performed in H9-NSCs, WT- and NPC-iNSCs and normalized to WT-hDFs using qRT-PCR. **(C)** Relative expression levels of endogenous factors (*SOX2* and *HMGA2*) and transgenes (pMX-SOX2 and pMX-HMGA2) were measured in WT- and NPC-iNSCs in comparison to H9-NSCs, whose expression is considered to be 1 for all genes using qRT-PCR. **(D)** Immunocytochemistry analysis of NSC-specific marker expressions from NPC-iNSCs (#3 and #12). **(E)** H9-NSCs, WT- and NPC-iNSCs were quantified according to NSC marker-positive cells over the total number of DAPI stained cells. *$P < 0.05$* P < 0.05.

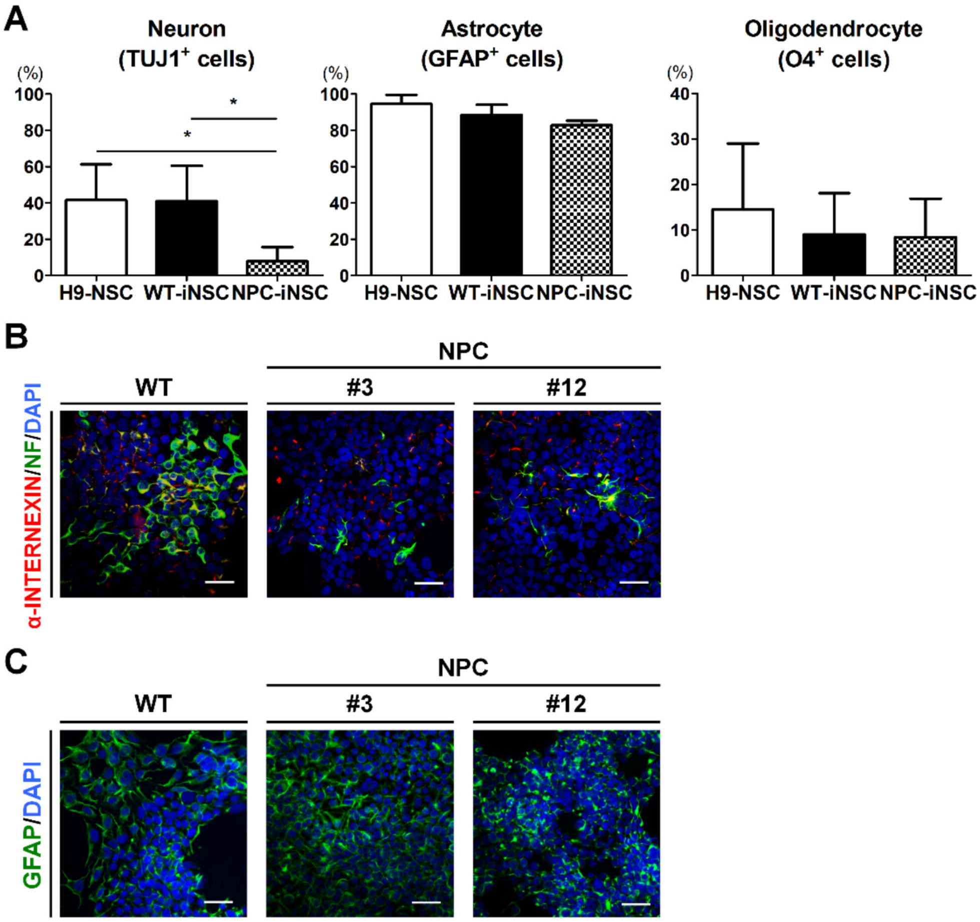

Supplementary Figure 2: Mutipotency of NPC-iNSCs. (**A**) Quantification of the differentiation efficiency of cells from H9-NSCs, WT- and NPC-iNSCs. (**B-C**) Immunocytochemistry analysis of differentiated WT- and NPC-iNSCs (#3 and #12) into neurons (B) and astrocytes (C). Nuclei were counterstained with DAPI, scale bar = 50 μm. *P < 0.05.

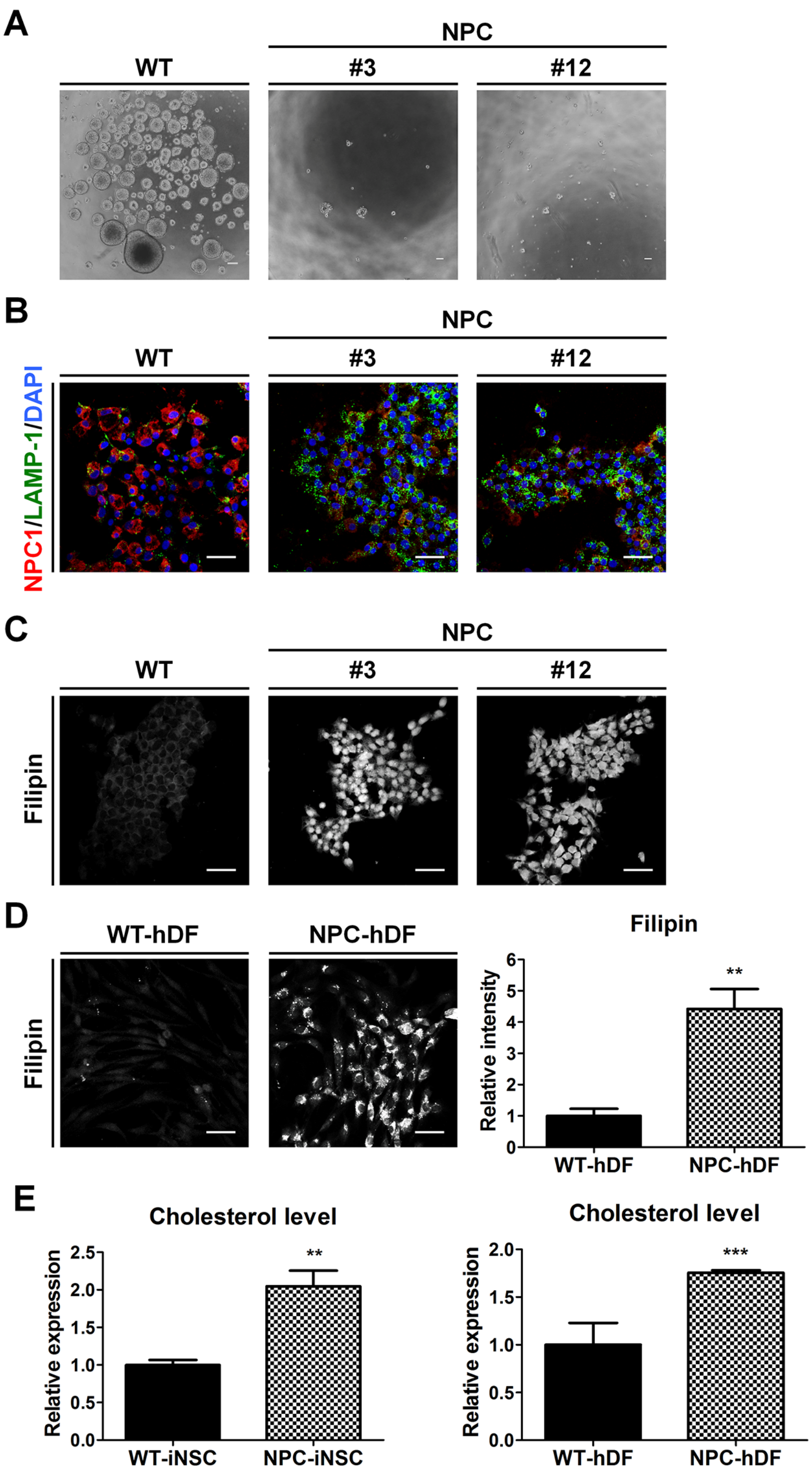

Supplementary Figure 3: NPC-hDFs exhibited massive cholesterol accumulation. **(A)** Phase-contrast images of neurospheres from WT- and NPC-iNSCs (#3 and #12), scale bar = 100 μm. **(B)** Immunocytochemistry analysis was performed in WT- and NPC-iNSCs (#3 and #12) using antibodies against NPC1 and LAMP-1. Nuclei were counterstained with DAPI, scale bar = 50 μm. **(C)** Unesterified cholesterol of WT- and NPC-iNSCs (#3 and #12) was detected by filipin staining, scale bar = 50 μm. **(D)** Filipin staining was performed to detect cholesterol accumulation in WT- and NPC-hDFs. The density of filipin-positive areas were quantified and the value of control was standardized as 1, scale bar = 50 μm. **(E)** Cholesterol levels of NPC-iNSCs/hDFs were quantified and normalized to WT-iNSCs/hDFs. $^{**}P < 0.01$, $^{***}P < 0.005$.

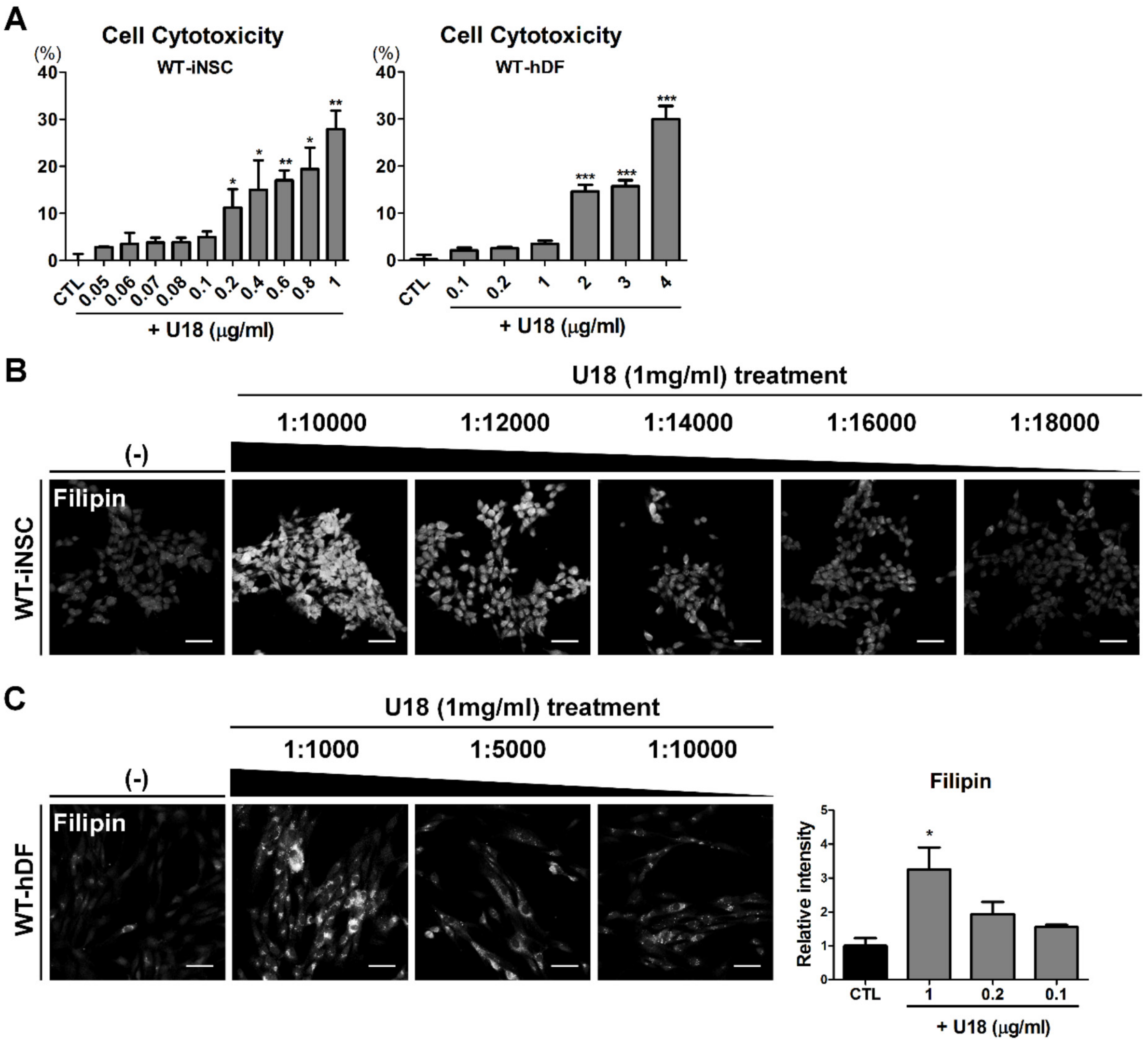

Supplementary Figure 4: U18-treated WT-iNSCs displayed abnormal cholesterol accumulation and impairments of self-renewal and neuronal differentiation. (**A**) The cytotoxicity of different range of U18 concentrations was tested in WT-iNSCs (left) and WT-hDFs (right). (**B-C**) U18 was treated at various concentration to WT-iNSCs (B) and WT-hDFs (C) to determine the appropriate efficacy of the reagent, detected by filipin staining, scale bar = 50 μm. $*P < 0.05$, $**P < 0.01$, $***P < 0.005$.

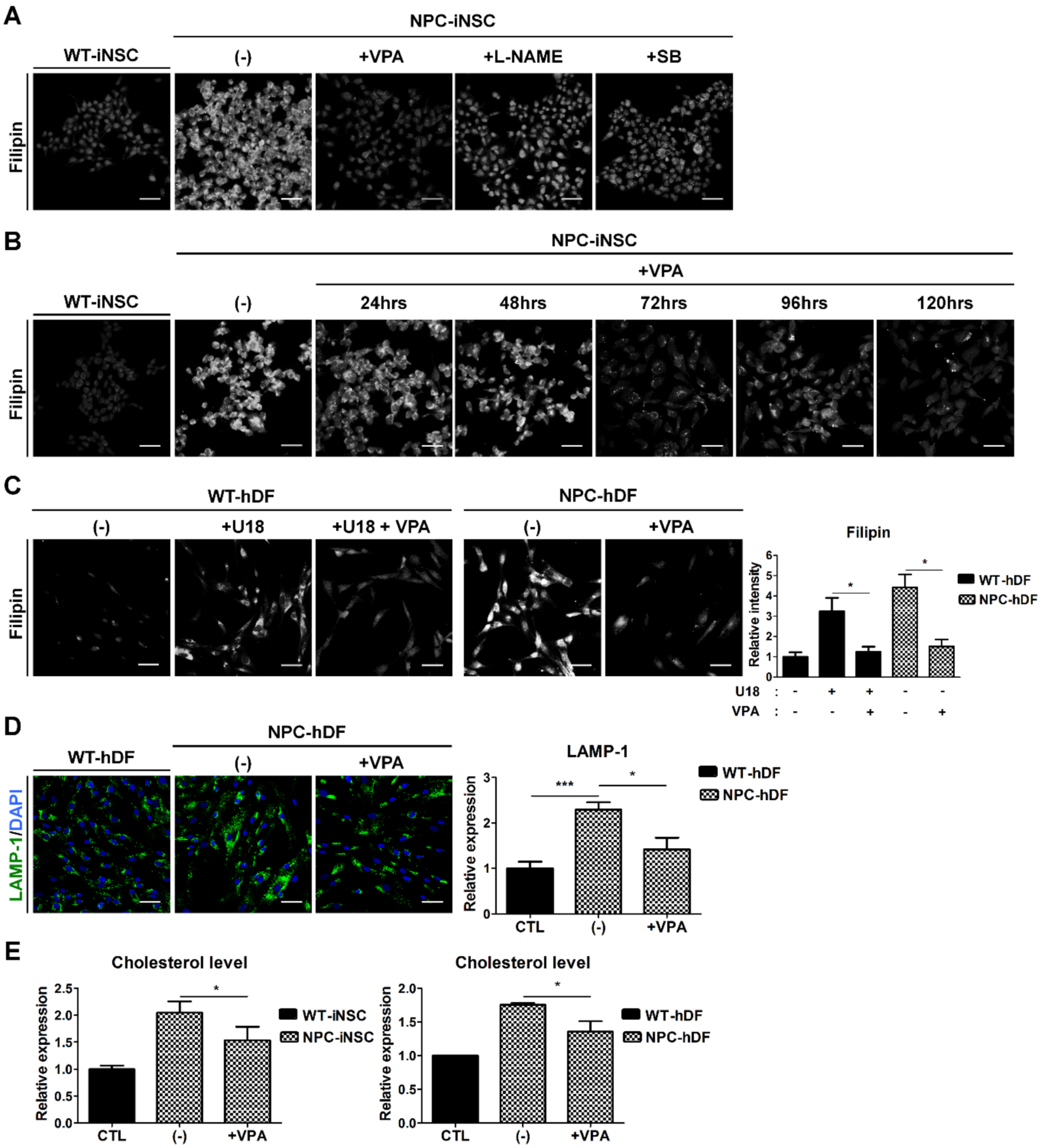

Supplementary Figure 5: VPA treatment restore the abnormal cholesterol accumulation and decrease the upregulated lysosomal marker expression. **(A)** Filipin staining was performed to detect cholesterol accumulation. NPC-iNSCs were treated with VPA, L-NAME, and SB202190, scale bar = 50 μm. **(B)** VPA was treated at various incubation time in NPC-iNSCs. Cholesterol accumulation was detected by filipin, scale bar = 50 μm. **(C)** VPA treatment had an effect on reduction of cholesterol accumulation in U18-treated WT- and NPC-hDFs, scale bar = 50 μm. **(D)** NPC-hDFs displayed decreased expression level of LAMP-1 after VPA treatment, scale bar = 50 μm. **(E)** Cholesterol levels of iNSCs and hDFs were quantified and normalized to WT. *$P < 0.05$, ***$P < 0.005$.

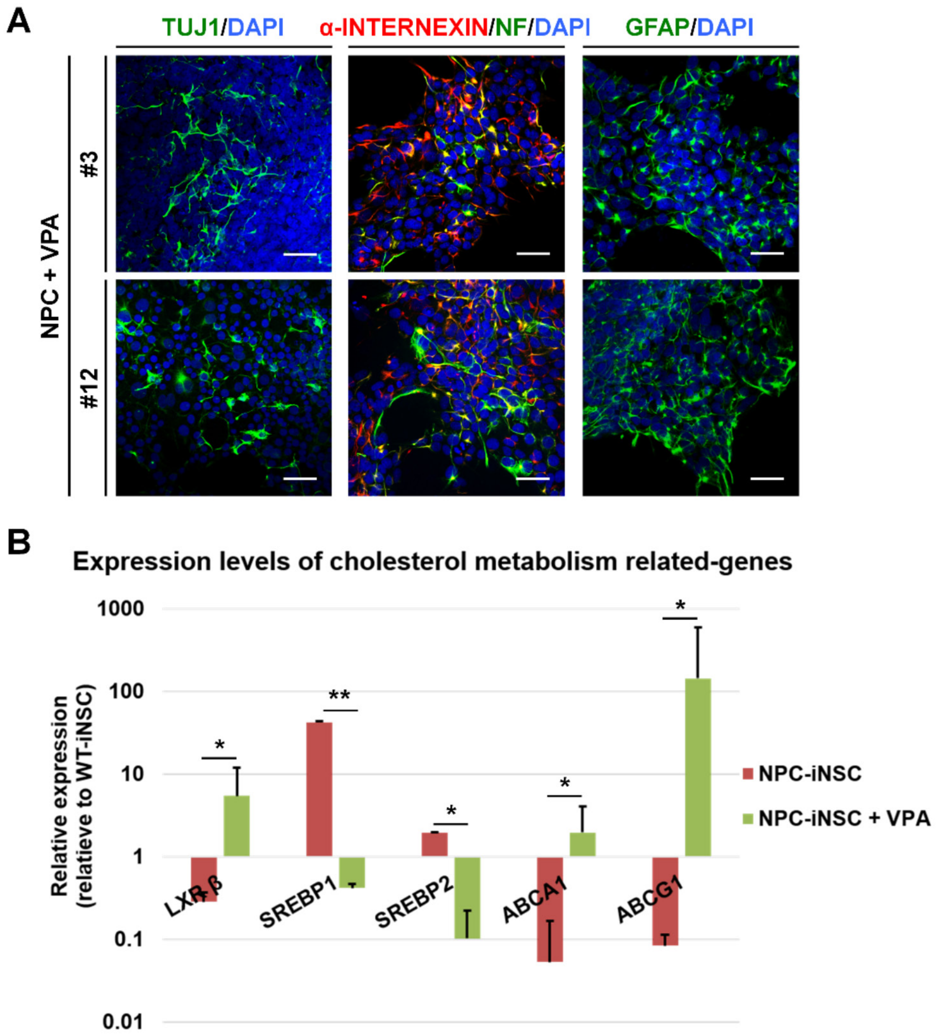

Supplementary Figure 6: VPA treatment enhances the neuronal differentiation in NPC-iNSCs and regulates the cholesterol metabolism. **(A)** NPC-iNSCs-derived from different donors were differentiated into neurons after VPA treatment, scale bar = 50 µm. **(B)** mRNA levels of *LXR β*, *SREBP1* and *2*, *ABC A1* and *G1* were examined using qRT-PCR. As a loading control, *GAPDH* were used. The expression levels of genes related to cholesterol metabolism were normalized to WT-iNSCs. *$P < 0.05$, **$P < 0.01$.
